# A Novel Benzofuran Derivative Moracin N Induces Autophagy and Apoptosis Through ROS Generation in Lung Cancer

**DOI:** 10.3389/fphar.2020.00391

**Published:** 2020-05-12

**Authors:** Chengcheng Gao, Xin Sun, Zhipan Wu, Huahua Yuan, Haote Han, Hongliang Huang, Yuhan Shu, Mengting Xu, Ruilan Gao, Shouxin Li, Jianbin Zhang, Jingkui Tian

**Affiliations:** ^1^College of Biomedical Engineering and Instrument Science, Zhejiang University, Hangzhou, China; ^2^Clinical Research Institute, Key Laboratory of Tumor Molecular Diagnosis and Individual Medicine of Zhejiang Province, Zhejiang Provincial People's Hospital, People's Hospital of Hangzhou Medical College, Hangzhou, China; ^3^Department of Oncology, Zhejiang Provincial People's Hospital, People's Hospital of Hangzhou Medical College, Hangzhou, China; ^4^School of Biosciences & Biopharmaceutics and Center for Bioresources & Drug Discovery, Guangdong Pharmaceutical University, Guangzhou, China; ^5^Institution of Hematology Research, The First Affiliated Hospital of Zhejiang Chinese Medical University, Hangzhou, China; ^6^Key Laboratory for Biomedical Engineering of Ministry of Education, Zhejiang-Malaysia Joint Research Center for Traditional Medicine, Zhejiang University, Hangzhou, China

**Keywords:** Moracin N, mitochondrial apoptosis, autophagy, mTOR, reactive oxygen species

## Abstract

**Introduction:**

The leaves of *Morus alba L* is a traditional Chinese medicine widely applied in lung diseases. Moracin N (MAN), a secondary metabolite extracted form the leaves of *Morus alba* L, is a potent anticancer agent. But its molecular mechanism remains unveiled.

**Objective:**

In this study, we aimed to examine the effect of MAN on human lung cancer and reveal the underlying molecular mechanism.

**Methods:**

MTT assay was conducted to measure cell viability. Annexin V-FITC/PI staining was used to detect cell apoptosis. Confocal microscope was performed to determine the formation of autophagosomes and autolysosomes. Flow cytometry was performed to quantify cell death. Western blotting was used to determine the related-signaling pathway.

**Results:**

In the present study, we demonstrated for the first time that MAN inhibitd cell proliferation and induced cell apoptosis in human non-small-cell lung carcinoma (NSCLC) cells. We found that MAN treatment dysregulated mitochondrial function and led to mitochondrial apoptosis in A549 and PC9 cells. Meanwhile, MAN enhanced autophagy flux by the increase of autophagosome formation, the fusion of autophagsomes and lysosomes and lysosomal function. Moreover, mTOR signaling pathway, a classical pathway regualting autophagy, was inhibited by MAN in a time- and dose-dependent mannner, resulting in autophagy induction. Interestingly, autophagy inhibition by CQ or Atg5 knockdown attenuated cell apoptosis by MAN, indicating that autophagy serves as cell death. Furthermore, autophagy-mediated cell death by MAN can be blocked by reactive oxygen species (ROS) scavenger NAC, indicating that ROS accumulation is the inducing factor of apoptosis and autophagy. In summary, we revealed the molecular mechanism of MAN against lung cancer through apoptosis and autophagy, suggesting that MAN might be a novel therapeutic agent for NSCLC treatment.

## Introduction

Lung cancer is the most common cancer with over 1.9 million newly diagnosed cases and leads to more than 1.7 million deaths worldwide in 2018 (Bray et al., 2018). Non-small-cell lung cancers (NSCLCs) account for about 85% of lung cancer, including adenocarcinoma, squamous cell carcinoma and large cell carcinoma (Relli et al., 2019). Lung cancer is often diagnosed at late stage due to the biomedical difficulties in detecting the cancer at early stage, leading to a lower five-year survival rate less than 15% (Corner et al., 2006; Molina et al., 2008; Keith and Miller, 2013). According to the the National Comprehensive Cancer Network (NCCN) evidence blocks V5 2019 of NSCLCs, chemotherapy is still the main treatment for advanced lung cancer, because of the limitation and dark side of other emerging treatments (Caino et al., 2015; Saâda-Bouzid et al., 2017; Champiat et al., 2018; Kim et al., 2019). Nevertheless, chemotherapy often leads to clinical drug resistance (Chung et al., 2012; Wang et al., 2019) and has serious side effect (Chang Y. S. et al., 2017). Thus, it is urgent to develop new effective therapeutic agents against lung cancer.

**Figure 1:**
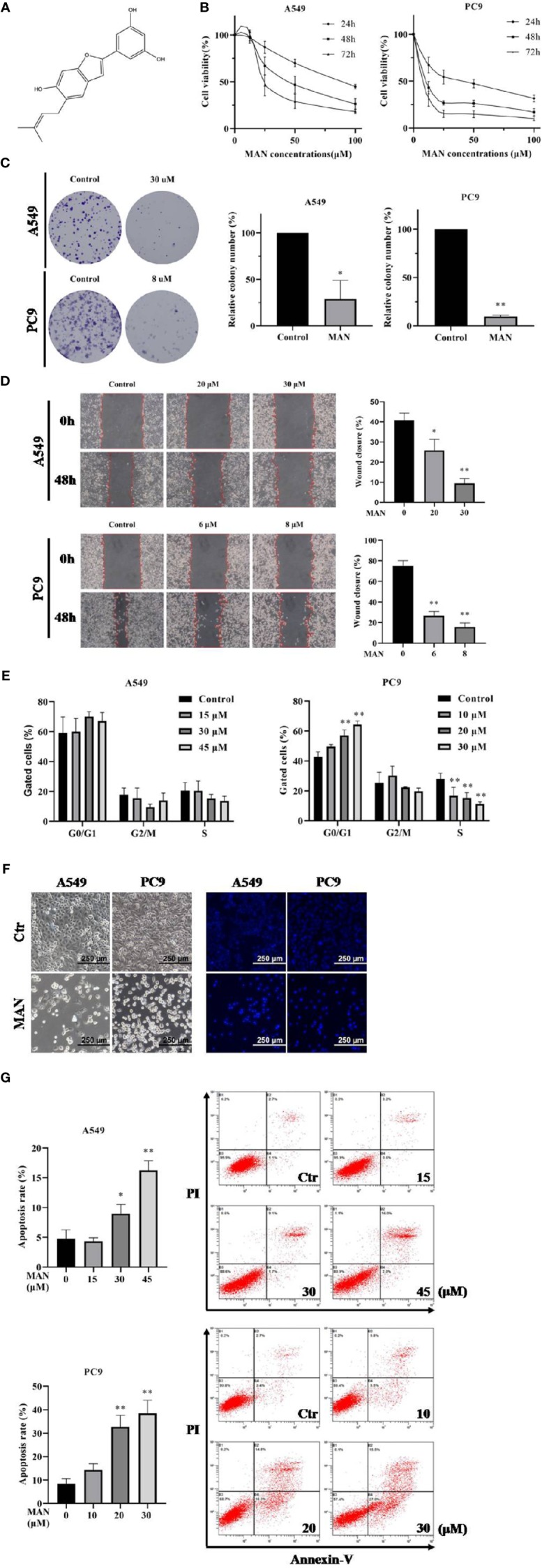
Moracin N (MAN) inhibits lung cancer cell proliferation. **(A)** MAN molecular structure. **(B)** A549 and PC9 cells were treated with various concentrations of MAN for 24 h, 48 h, and 72 h. Cell viability was detected by MTT assay. **(C)** Cells were treated with MAN (30 μM or 8 μM) for 48 h. Then cells were collected and reseeded into 6-well plates with a density of 500 cells per well for another 14 days to form clonies. The number of clonies were counted by Image J and statistically analyzed. *p < 0.05 ** *p* < 0.01. **(D)** Cells were treated with various concentrations of MAN for 48 h and the scratch was draw by pipette tip. Then cells were cultured in medium containing 2.5% FBS. The wound healing area was measured by photoshop. * *p* < 0.05 ** *p* < 0.01. **(E)** Cells were treated with various concentrations of MAN for 48 h. Then cells were collected and the cell cycle were detected by flow cytometry using cell cycle analysis kit. ** *p* < 0.01. **(F)** Cell and nuclear morphology were observed after 48 h MAN (A549: 30 μM, PC9: 10 μM) treatment by optical and fluorescence microscope, respectively. Cell nucleus was stained by Hoechst 33342 (10 μg/ml). **(G)** Apoptosis rates were detected by flow cytometry. Cells were treated with various concentrations of MAN for 48 h. Then cells were collected and stained by the apoptosis analysis kit according to manufacturer's protocol. Both Annexin V^+^/PI^-^ and Annexin V^+^/PI^+^ cells were regarded as the apoptotic cells. *p <0.05 ** *p*< 0.01.

Natural compounds from chinese medicine are the origins of some anti-cancer drugs (Gupta et al., 2001; Diederich and Cerella, 2016). The leaves of *Morus alba* L is a traditional Chinese medicine used for lung diseases. Previous research has proved the anti-cancer and anti-inflammatory effect of the methylene chloride extracts of the leaves of *Morus alba* L (Park et al., 2012; Min et al., 2019). For example, Moracin M can inhibit inflammatory responses through inhibition of mTOR pathway (Guo et al., 2018). Here, we extracted one secondary metabolite from the leaves of *Morus alba* L as described (Gu et al., 2010; Hu et al., 2017) with its structure 5-[6-hydroxy-5-(3-methylbut-2-en-1-yl)-1- benzofuran-2-yl]benzene-1,3-diol (Moracin N, MAN, **Figure 1A**). Pharmacological studies show the broad biological activities of MAN, including tyrosinase inhibition, anti-virus, anti-oxidant and anti-liver cancer (Zheng et al., 2010; Hu et al., 2017; Tu et al., 2019). However, there is little study on the effect of MAN on lung cancer.

Autophagy is a conserved intracellular self-digestion by lysosomes, including macroautophagy, microautophagy and chaperone-mediated autophagy (Klionsky and Emr, 2000; Mizushima et al., 2008). During macroautophagy, a double-membrane cytosolic vesicle named autophagosome selectively and/or non-selectively sequestrates cargoes, including cytoplasm, organelles or microbes, which are fused with lysosomes and degraded (He and Klionsky, 2009). Generally, autophagy is considered to be the protective mechanism in cells through maintaining cellular homeostasis in response to stresses (Levine and Kroemer, 2008). However, the role of autophagy in cancer is complicated and serves as a double-edged sword (Acharya et al., 2011; Kubisch et al., 2013; Pan et al., 2014; Sharma et al., 2014; Mi et al., 2016; Datta et al., 2019). In terms of lung cancer, some researches demonstrate that autophagy contributes to cell survival and autophagy inhibition can reverse multi-drug resistance (Pan et al., 2014; Mi et al., 2016; Datta et al., 2019), while other researches show that some compounds, including naphthazarin vitamin D and its analog EB 1089, induce cytotoxic autophagy to enhance the therapeutic efficacy (Acharya et al., 2011; Sharma et al., 2014). Thus, it is necessary to clarify the role of autophagy in lung cancer and develop novel therapeutics targeting autophagy.

Apoptosis, including extrinsic apoptosis and intrinsic apoptosis, is a programmed cell death characterized by caspase activation, cell membrane valgus and chromatin condensation, et al. (Sawada et al., 2000; Elmore, 2007; Kantari and Walczak, 2011; Kalimuthu and Se-Kwon, 2013). Mitochondria is not only the intracellular energy factory, but also involved in cell apoptosis (Bhola and Letai, 2016; Saki and Prakash, 2017; Banoth and Cassel, 2018). When mitochondria is dysfunctional, mitochondria fissions into separate units. With its membrane potential disrupted, cytochrome c is released into cytosol and caspase cascade is activated, leading to the intrinsic apoptosis (Tait and Green, 2010). Previous research has established that apoptosis is an important target for NSCLC therapy (Pan et al., 2014; Mi et al., 2016; Datta et al., 2019). Hence, we attempted to determine the role of mitochondrial apoptosis in MAN against lung cancer.

In this study, we aimed to examine the anti-cancer effect of MAN on human NSCLC and reveal the underlying molecular mechanism. We found that MAN inhibits human lung cancer cell growth by inducing mitochondrial apoptosis and autophagy. MAN treatment causes ROS generation, which further activates apoptosis and autophagy. Interestingly, the impairment of autophagy attenuated MAN-caused cell death, suggesting that autophagy serves as cell death. This is the first study to reveal the molecular mechanism of MAN against human lung cancer. And our findings demonstrate the great potential of MAN in the treatment of human NSCLC.

## Materials and Methods

### Cell Culture

A549 and PC9 cells were obtained from American Type Culture Collection (ATCC). Hela cells stably expressing GFP-LC3 or L929 cells stably expressiong RFP-GFP-LC3 were kindly provided by Prof. Shen Han-Ming (National University of Singapore, Singapore). A549 and PC9 cells were maintained in RPMI 1640 medium (Life Technologies, 22400-089) containing 10% fetal bovine serum (Sangon Biotech, Shanghai). Hela and L929 cells were maintained in DMEM with 10% fetal bovine serum (HyClone, SV30160.03). Cells were incubated in cell culture incubator with 5% CO_2_ at 37°C.

### Reagents and Antibodies

The antibodies used in the present study were purchased from Cell Signaling Technology, including Bcl-2, Bax, caspase 3, β-actin, LC3, AKT, phospho-AKT (Ser473), mTOR, phospho-mTOR (Ser2448), S6 ribosomal protein, phospho-S6 ribosomal protein (Ser235/236) and ATG5, with their catalog number D17C4, D2E11, D3R6Y, E4D9Z, D11, 11E7, D9E, 7C10, D9C2, 54D2, D57.2.2E, and D5F5U, respectively. Other antibodies included cytochrome c (M1701-9), goat anti-rabbit/mouse IgG-HRP (ImmunoResearch Laboratories) and FITC goat anti-mouse IgG (Beyotime, A0562).

Reagents used in our research included: cell cycle and cell apoptosis analysis kits (KEYGEN, KGA511 and KGA107); Hochest33342 staining kit (Beyotime, C1025); mitochondrial membrane potential detection kit and ROS measurement kit (NJJCBIO, G009-1-3 and E004-1-1), MitoTracker Deep Red FM (Life Technologies, M22426), Lyso-Tracker Red (Invitrogen, L7528), cholorquine (CQ, PubChem, 2719), 3-MA, and N-acetylcysteine (NAC) (aladdin, M129496 and A105422). Moracin N (MAN) was isolated and purified by Prof. Tian Jingkui from the Key Laboratory of Biomedical Engineering at Zhejiang University.

### Cell Viability Analysis

Cell viability was measured using 3-(4,5-dimethylthiazol-2-yl)-2,5-diphenyltetr-azolium bromide (MTT, Solarbio, M8180) assay. Cells were first seeded into 96-well plates in a final volume of 100 μl (3,000 cells per well) for 24 h and then treated with different concentrations of MAN (0~80 μM) for 24 h, 48 h, or 72 h. After treatment, 50 μl MTT (2.5 mg/ml) solution was added into each well and incubated for another 2h. Finally, we replaced the medium of each well with 200 μl DMSO. The plates were shaken for 3 min in a microplate reader and the optical density was measure at 570 nm. The relative cell viability rate = (average optical density of experimental group/average optical density of control group) ×100%.

### Cell Cycle Analysis

Cells were seeded into 6-well plates (3×10^5^ cells per well) overnight and treated with MAN (0~45 μM). After treatment, cells were harvested and resuspended with 1 ml 70% ice-cold alcohol stored at 4°C overnight. Then, cells were washed twice with PBS and cultured with RNase A at 37°C for 30 min and stained the cells with propidium iodide. Cell cycle phases were analyzed by flow cytometry.

### Clony Formation Assay

Cells were seeded into 6-well plates (3×10^5^ cells per well) overnight. After MAN treatment, the medium was removed and cells were washed twice with PBS. Then, cells were collected and reseeded into 6-well plates with a density of 500 cells per well for another 14 days. When the size of cell clonies were bigger, the medium was reomved and cells were stained with 0.1% crystal violet for 30 min. The number of clones were counted by Image J.

### Cell Apoptosis Analysis

Annexin V-FITC/PI apoptosis detection kit (KeyGEN BioTECH, KGA105) was used for cellapoptosis analysis. Briefly, cells were seeded into 6-well plates (3×10^5^ cells per well) and cultured for 24 h, followed by the treatment of different concentrations of MAN (0∼ 45 μM). Then, cells were collected after washing twice with cold PBS. Cells were resuspended with 500 μl binding buffer with 5 μl Annexin V-FITC and 5 μl of propidium iodide. The samples were analyzed using flow cytometry (Cytoflex, Beckman) and the percentage of apoptotic cells were calculated by the internal software system of Cytoflex.

### Hochest Staining

Cells were first treated with MAN for 48 h and then washed twice with PBS. Hochest 33,342 dyeing liquid (Beyotime) was added for 30 min staining. Then, cells were washed softly with PBS and observed under fluorescence microscope.

### Western Blotting

Cells were seeded into 6cm dishes (5×10^5^ cells per dish) overnight and treated with MAN (0∼ 45 μM). After treatment, cells were collected by scraping and washed twice with PBS. Then, cells were lysed with RIPA buffer (50 mM Tris-HCl pH 7.4, 150 mM NaCl, 1% Triton X-100, 1% sodium deoxycholate, 0.1% SDS, and sodium orthovanadate, sodium fluoride, EDTA, leupeptin). Protein concentrations in the supernatant were determined by a BCA Protein Assay Kit (Solarbio, PC0020). Equal amounts of proteins were separated by SDS-polyacrylamide gels and then electroblotted onto polyvinylidene fluoride (PVDF, Bio-Rad, 1620184) membrane. The membranes were blocked with TBST plus 5% skimmed milk for 2 h and incubated with primary antibodies (1:1,000) overnight at 4°C. After washed three times with TBST, the membranes were incubated with secondary antibodies (1:5,000) for 1 h at room temperature. Before development, the membranes were washaed three times again and the immunoblots were visualized with an ECL system.

### Mitochondrial and Lysosomal Morphology

Cells were seeded into petri dish with glass bottom and treated with MAN for 48 h. Then, the cells were stained with mitotracker deep red or lysotracker red for 30 min. Confocal microscope was used to observe the mitochondrial and lysosomal morphology. The mean mitochondrial branch length was calculated by Image J.

### Mitochondrial Memberane Potential

Cells were treated with MAN for 48 h. JC-1 probe (KeyGen, Nanjing, China) was added into the cells for 30 min staining. Then the fluorescence was observed by confocal microscope or flow cytometry.

### ROS Measurement

The ROS levels were measured using a fluorescent dye 2'7'-dichlorfluorescein diacetate (DCFH-DA, Beyotime, S0033). Cells were seeded into 6-well plates (3x10^5^ cells per well) and cultured for 24 h. After MAN treatment, the medium was removed and cells were washed with PBS and then cultured in serum-free medium containing 10 μM DCFH-DA for 30 min. Finally, the cells were collected for flow cytometry analysis.

### Immunofluorescence Staining

Cells were seeded into petri dish with glass bottom with a suitable density. After MAN treatment, cells were fixed with 4% paraformaldehyde followed with permeabilization by 0.24% Triton X-100. Cells were first incubated with different antibodies overnight and then incubated with flurochrome-conjugated secondary antibody for another 1 h. Cell fluorescence was observed under confocal microscope and photographed.

### Confocal Microscope Assay

Autophagosome formation assay was conducted using Hela cells with GFP-LC3 stably expressing or L929 cells with RFP-GFP-LC3 stably expressing. Briefly, cells were seeded into glass slides. After MAN treatment, cells were examined under confocal microscope. The puncta number was calculated by Image J and the co-localization coefficient of GFP-LC3 puncta and lysosomes was analysed by Image pro plus.

### Small Interfecting RNA(siRNA) and Transient Transfection

Cells were firstly seeded into 6-well plates overnight and then siRNA targeting Atg5 was transfected into A549 cells using Lipofectamine 2000 according to the manufacturer's protocol. After 72 h, cells were harvested and reseeded into plates for different treatment.

### Transmission Electron Microscope (TEM)

A549 cells were seeded into 5cm petri dish followed with MAN treatment. Then cells were collected with scraper and centrifuged at 4°C with 2,000 rpm. The precipitated cells were fixed with 4% glutaraldehyde for another 2 h at room temperature and saved at 4°C. Electron photomicrographs of A549 cells were taken by Wuhan Goodbio Technology Co.

### Statistical Analysis

All experiments were repeated for three times. The results were analyzed using one-way ANOVA for statistical significance. Data were expressed as the mean ± standard error. * *p* < 0.05, ** *p* < 0.01, *** *p* < 0.001. As long as *p*-value < 0.05, the difference was considered statistically significant.

## Results

### MAN Inhibits Lung Cancer Cell Proliferation

MAN (**Figure 1A**) was isolated from the leaves of *Morus alba* L as a brown powder with a relative molecular mass of 310 g·mol^-1^. The ^1^H-NMR spectrum was as follows: δ_H_7.09 (1H, s, H-4), 6.79 (1H, s, H-7), 6.76 (1H, s, H-3), 6.65 (1H, s, H-2'), 6.64 (lH,s, H-6'), 6.13 (1H, t, J=4.3, 2.2Hz, H-4'), 5.26 (1H, t, J=2.8, 1.4Hz, H-9), 3.25 (2H, m, H-8), 1.65 (3H, s, H-11), and 1.63 (3H, s, H-12). The ^13^C NMR spectrum was as follows:δ_C_ 18.2 (C-ll), 26.4 (C-12), 29.9 (C-8), 98.3 (C-7), 102.7 (C-3), 103.8 (C-4'), 104.3 (C-2', c-6'), 121.8(C-4), 123.2 (C-4a), 124.8 (C-8), 126.6 (C-6), 133.3 (C-10), 134.4 (C-1') 155.0 (C-6), 155.9 (C-7a), 156.2 (C-2), and 1,660.3 (C-3', C-5').

To investigate the cytotoxicity of MAN in lung cancer, NSCLC cells PC9 and A549 were treated with various concentrations of MAN for 24 h, 48 h, and 72 h. Using the MTT assay, we observed a time- and dose-dependent decrease in the values, indicating the inhibition of cell growth. IC50 in A549 and PC9 cells were 48.4 μM and 6.6 μM, respectively (**Figure 1B**). The ability of cells to survive when exposure to MAN was also determined by clony formation assay. As seen in **Figure 1C**, MAN significantly reduced the number of clonies either in A549 or PC9 cells. Further, wound-healing assay was performed to explore the effect of MAN on cell migration. The results showed that MAN treatment inhibited PC9 and A549 cells migration in a dose-dependent manner (**Figure 1D**).

The effect of MAN on cell proliferation may be different between A549 and PC9 cells. According to cell cycle analysis, MAN increased the percentage of PC9 cells in the G0/G1 phase while it had no effect on A549 cells (**Figure 1E**). In addition, the MAN-treated cells exhibited morphological changes, including cell shrinkage and nuclear chromatin condensation, indicating the possibility of apoptosis (**Figure 1F**). The Annexin V and PI staining was performed to verify the apoptosis, in which MAN treatment significantly increased the apoptotic cell percentage (Annexin V^+^ cells) either in A549 or PC9 cells in a dose-dependent manner (**Figure 1G**).

### MAN Triggeres Mitochondrial Apoptosis in Lung Cancer Cells

**Figure 2:**
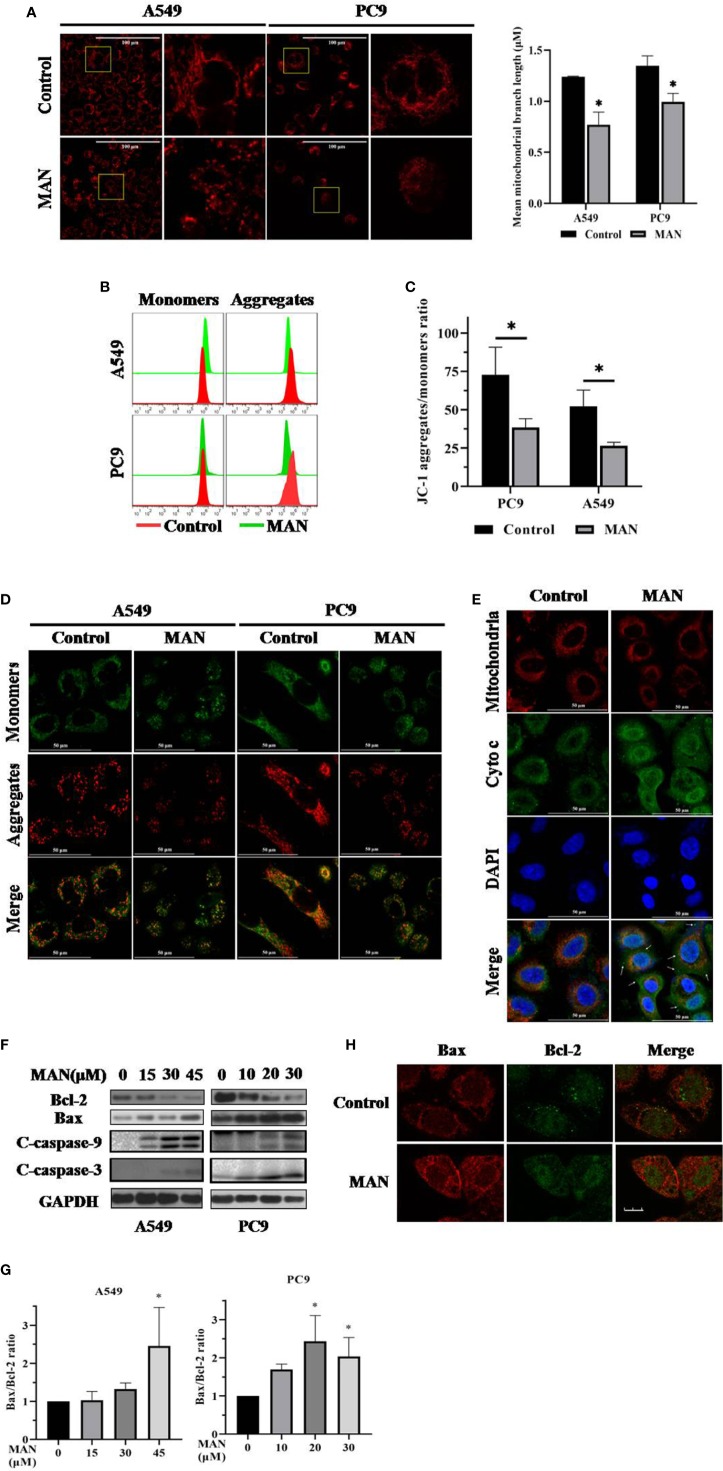
Moracin N (MAN) treatment leads to mitochondrial dysfunction. **(A)** Cells were treated with MAN (A549: 30 μM, PC9: 10 μM) for 48 h followed by loading with MitoTracker Deep Red (100 nM) for 30min. The mitochondrial morphology was observed by confocal (scale bar 25 μm). Mean mitochondrial branch length was calculated by Image J in cells. * *p* < 0.05. **(B–D)** Cells were treated with MAN (A549: 30 μM, PC9: 10 μM) for 48 h. After staining with JC-1 probe, cell fluorescence was examined by flow cytometry and confocal microscope. *p <0.05. **(E)** A549 cells were treated with MAN (30 μM) for 48 h. Then, cells were stained with MitoTracker Deep Red followed with immunostaining with cytochrome c. The colocalization of mitochondria and cytochrome c was detected by confocal microscope. **(F)** A549 and PC9 cells were firstly treated by various concentrations of MAN (0~45μM) for 48 h. Then, cells were harvested for western blotting to examine the apoptosis-related proteins levels. β-actin was used as loading control. **(G)** as in (**F**), the Bax/Bcl-2 ratio was measured by photoshop. * *p* < 0.05. **(H)** Immunostaining of Bax (red) and Bcl-2 (green) in A549 cells under MAN treatment (scale bar 10μm).

Mitochondria plays a pivotal role in cancer intracellular signaling in response to drug treatment (Guerra et al., 2017). In the present study, we aimed to investigate the mitochondrial status after MAN treatment. As shown in **Figure 2A**, mitochondria was fragmented into smaller units in MAN-treated cells when compared with untreated cells. Statistical analysis showed that MAN significantly decreased the mitochondrial branch length in cells (**Figure 2B**). Meanwhile, we used JC-1 dye to detect mitochondrial member potential (MMP). JC-1 is a cationic dye that accumulates in energized mitochondria. It is predominantly a monomer yielding green flurescence at low MMP while it aggregates yeilding red colored emission at high MMP. Thus, the aggregate/monomer ratio can reflect the MMP. JC-1 staining showed that the ratio of JC-1 aggregates to JC-1 monomers decreased, representing the lower MMP under MAN treatment (**Figures 2B–D**). All of these results indicated the mitochondrial dysfunction in MAN-treated cells.

Mitochondrial dysfunction usually results in mitochondrial apoptosis. Next, we determined the localization of cytochrome c and found that the localization of cytochrome c in mitochondria was significantly decreased and cytochrome c was released into cytoplasm in MAN-treated cells (**Figure 2E**), indicating the mitochondrial apoptosis. In addition, the levels of anti-apoptotic pretein Bcl-2 and pro-apoptotic pretein Bax, which regulates the release of cytochrome c (Ashkenazi, 2008), were also examined. The Bax/Bcl-2 ratio was significantly increased in a dose-dependent manner under MAN treatment (**Figures 2F, G**), while their interaction was decreased by MAN (**Figure 2H**).

### MAN Induces Autophagy by Inhibiting the AKT/mTOR Pathway

**Figure 3:**
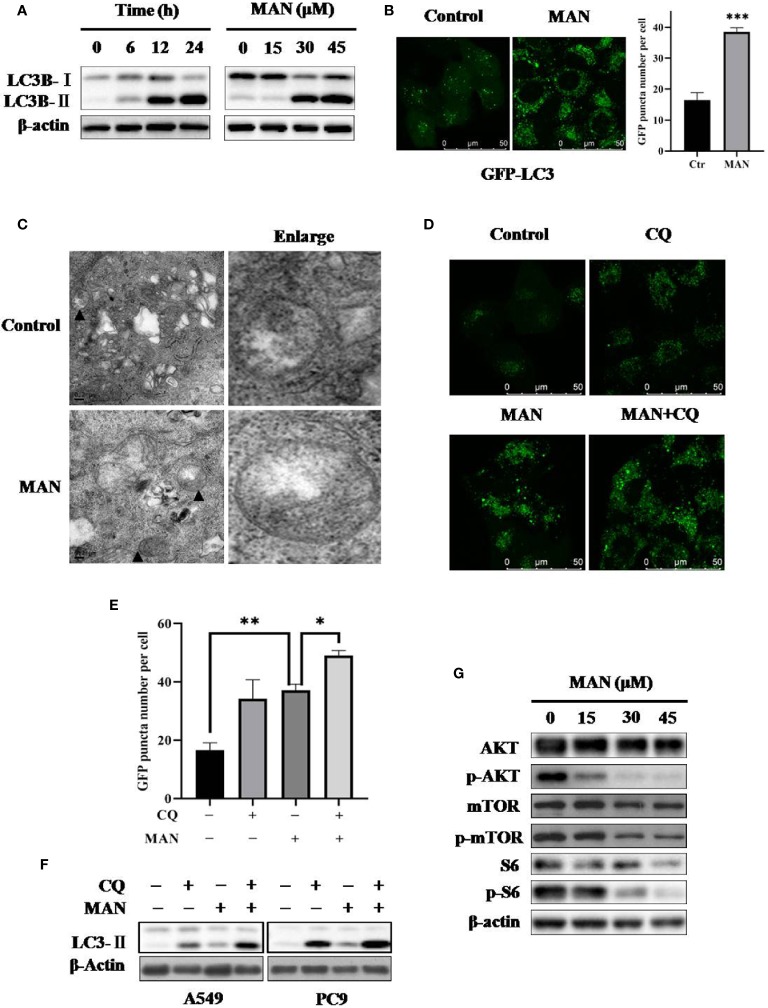
Moracin N (MAN) induces autophagy by inhibiting the AKT/mTOR pathway. **(A)** A549 cells were treated with MAN with various concentrations (0~45μM) for different time points (6 h, 12 h, and 24 h). Then cells were harvested for western blotting to detect LC3 protein levels. β-Actin was used as loading control. **(B)** GFP-LC3 expressing Hela cells were treated with MAN (20 μM) for 24 h and the GFP-LC3 puncta was examined using confocal microscopy (scale bar 50 μm). GFP puncta quantity was calculated by Image J. *** *p* < 0.001. **(C)** TEM images of the ultrastructure of A549 cells under MAN treatment (scale bar 0.2 μm). ▲ refer to double-membrane autophagosomes. **(D–F)** Hela cells with GFP-LC3 stably expressing, A549 or PC9 cells were treated with MAN (20 μM, 30 μM, and 15 μM respectively) with or without CQ (10 μM) for 48 h. Then, LC3 protein levels were detected by western blotting with β-actin used as loading control. The GFP-LC3 green puncta was detected by confocal microscope and quantified by Image J. * *p* < 0.05. ** *p* < 0.01. **(G)** as in (A), the AKT/mTOR pathway related proteins were examined by western blotting and β-actin was used as a loading control.

Autophagy is essential for cell survival and death. LC3 protein is the marker of autophagosome formation, which is dispersed in the cytoplasm in the form of LC3-I and is aggregated on the autophagosome membrane when transformed to LC3-II (Ni et al., 2011). To examine the effect of MAN on autophagy, the autophagy marker LC3 was determined under MAN treatment. As shown in **Figure 3A**, MAN treatment increased LC3-II levels in a time- and dose-dependent manner. Besides, the formation of GFP-LC3 puncta also demonstrated a dramatic aggregation of LC3-II in MAN-treated cells (**Figure 3B**). Moreover, the ultrastructure of A549 cells showed that there were double-membrane autophagosomes in MAN-treated cells (**Figure 3C**). From our perspective, autophagy induction specifically refers to an increase in autophagic flux rather than simply an increase of autophagic markers in cells. Thus, we investigated the autophagy flux using chloroquine (CQ), a lysosomal inhibitor. Under confocal microscopy, the GFP-LC3 puncta was further increased in MAN plus CQ treated cells when compared with MAN treatment alone (**Figures 3D, E**), suggesting the enhanced autophagy flux. In addition, the similar result was also observed in MAN-treated A549 and PC9 cells (**Figure 3F**), confirming the induction of autophagy.

To reveal the molecular mechansim, we determined the changes of the AKT/mTOR signaling pathway, a classical pathway regualting autophagy. Western blotting results showed that MAN treatment decreased the phosphorylation levels of AKT (ser473), mTOR (ser2448) and S6 ribosomal protein (ser235/236), a downstream protein of mTOR, in a dose- and time-dependent manner (**Figure 3G**), indicating that inhibition of the AKT/mTOR pathway is responsible for MAN-induced autophagy.

### MAN Activates the Lysosomal Function and Promotes the Fusion of Lysosomes and Autophagosomes

**Figure 4:**
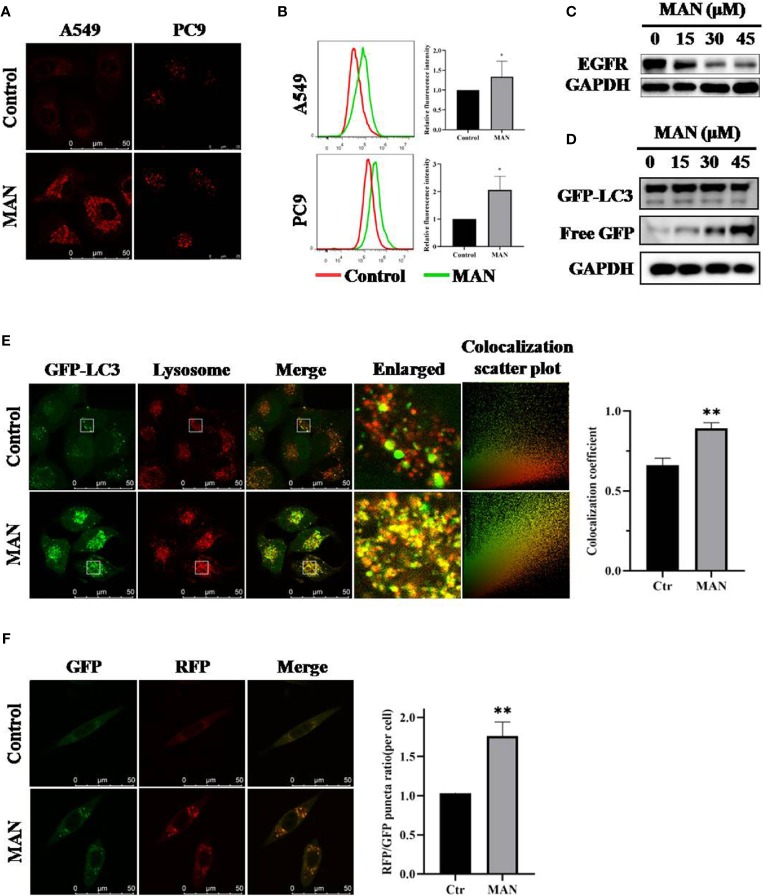
Moracin N (MAN) activates lysosomal function and promotes the fusion of lysosomes and autophagosomes. **(A**, **B)** A549 and PC9 cells were treated with MAN (30 μM and 20 μM, respectively) for 24 h followed by staining with Lysotracker Red (50 nM) for 30 min. Then, lysosomes were observed by confocal microscope (scale bar 50 μm) and the fluorescence intensity was measured by flow cytometry. * *p* < 0.05. **(C, D)** A549 or GFP-LC3 expressing Hela cells were treated with various MAN concentrations (0~45μM) for different time points (6 h, 12 h, and 24 h). Then, cells were harvested for western blotting to detect EGFR and free GFP level. β-actin was used as a loading control. **(E)** Hela cells with stably expressing GFP-LC3 were treated with MAN (15 μM) for 24 h followed by Lysotracker Red (50 nM) staining for 30 min. The cells were observed under confocal microscope (scale bar 50 μm). The colocalization scatter plot and colocalization coefficient was detected using Image Pro Plus. ** *p* < 0.01. **(F)** L929-tfLC3 cells were firstly treated with MAN (10 μM, 24 h) and then the cells were photographed using confocal microscope (scale bar 50 μM). Image J was applied to count GFP and RFP puncta number and the RFP/GFP ratio was calculated. ** *p* < 0.01.

Autophagosomes fusion with lysosomes followed by degradation represents the completion of autophagy (Davidson and Vander Heiden, 2017). To assess the effect of MAN on the lysosome, we stained A549 and PC9 cells with LysoTracker Red after MAN treatment. Under confocal microscope, we observed more enlarged lysosomes in MAN-treated cells with brighter red puncta (**Figure 4A**), indicating the enhancement of lysosomal acidification. This was further verified by flow cytometry, which showed an increase in red fluorescence (**Figure 4B**). In addition, we examined the protein levels of EGFR (epidermal growth factor receptor), a substrate degraded by the lysosome (Beaumont et al., 2018; Liu et al., 2018c; Mauthe et al., 2018; Zhang et al., 2018). The results in the **Figure 4C** showed that MAN accelerated the degradation of EGFR with doses, indicating the activated lysosomal degradative function. We also conducted western blotting to measure the cleavage of GFP-LC3, which is degraded within autolysosomes and regarded as an autophagic degradation assay. As shown in **Figure 4D**, free GFP levels were increased with MAN treatment, indicating the enhanced lysosomal degradation.

Lastly, we determined the effect of MAN on autolysosome formation. Cells with GFP-LC3 stably expressing were stained with LysoTracker Red after MAN treatment. Confocal results showed that MAN treatment significantly increased the overlay of GFP-LC3 puncta and the lysosome (**Figure 4E**), suggesting the enhanced fusion of autophagosomes and lysosomes. In addition, in MAN-treated L929 cells, a cell line stably expressing mRFP-GFP tandem fluorescent-tagged LC3B (tfLC3), in which the RFP component was stable while GFP could be degraded in acidic and proteolytic environment, RFP-only (RFP^+^/GFP^-^) puncta and the RFP/GFP ratio increased when compared with untreated cells (**Figure 4F**), confirmed the enhanced fusion between autophagosomes and lysosomes.

### MAN Induces Apoptosis and Autophagy Through ROS Accumulation

**Figure 5:**
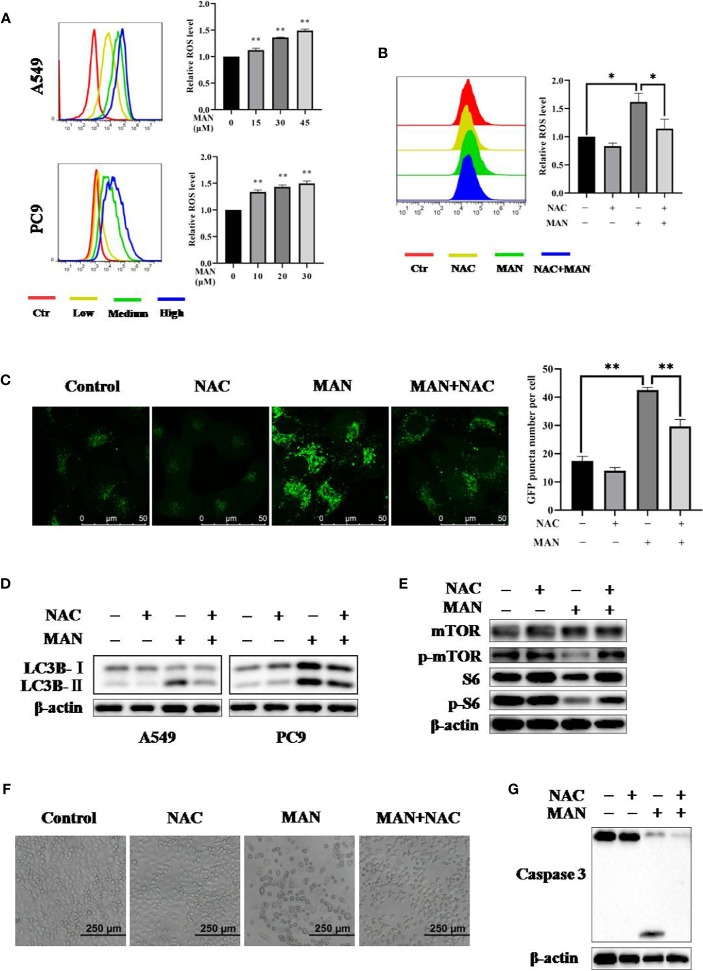
Moracin N (MAN) induces apoptosis and autophagy through reactive oxygen species (ROS accumulation). **(A)** A549 and PC9 cells were treated with MAN (A549: 15, 30, and 45 μM. PC9: 10, 20, and 30 μM) for 48 h. Then, cells were loaded with DCFH-DA probe to labeling intracellular ROS. Fluorescence intensity was measured by flow cytometry. ** *p* < 0.01. **(B)** as in (A), A549 cells were treated with MAN (30 μM) with or without NAC (5 mM) for 24 h. Then, cells were stained by DCFH-DA probe to detect intracellular ROS using flow cytometry. * *p* < 0.05. **(C, D)** GFP-LC3 expressing Hela cells, A549 or PC9 cells were treated by MAN (15 μM, 30 μM, and 20 μM) with or without NAC (5 mM) for 24 h. The GFP-LC3 puncta was examined by confocal microscope (scale bar 50 μm) and GFP-LC3 puncta number was counted by Image J. ** *p* < 0.01. LC3 protein levels were measured using western blotting. β-actin was used as a loading control. **(E)** as in (D), A549 cells were harvested after treatment and the AKT/mTOR pathway related proteins as well as caspase 3 were examined by western blotting. β-actin was used as loading control. **(F, G)** A549 cells were treated by MAN (30 μM) with or without NAC (5 mM) for 48 h. Cell morphology was observed by optical microscope and cells were harvested for western blotting.

ROS can regulate various singnaling pathways, including apoptosis and autophagy (Geng et al., 2017; Liu et al., 2018c). Hydrogen peroxide is the primary ROS in cells (Liu et al., 2018c). Flow cytometry results showed that MAN dramatically increased ROS generation in a dose-dependent manner either in A549 or PC9 cells (**Figure 5A**). Then, we applied N-acetyl cysteine (NAC), which is an anti-oxidant, to reduce oxidative stress. As expected, NAC reduced ROS generation in MAN-treated cells (**Figure 5B**), suggesting that MAN induces ROS accumulation in lung cancer cells.

Next, to assess the relationship between ROS and MAN-induced autophagy, cells with stably expressing GFP-LC3 were treated with MAN in the presence of NAC. As shown in **Figure 5C**, NAC treatment abolished the increase of GFP-LC3 puncta after MAN treatment, indicating that high levels of ROS are the cause of autophagy. The western blotting results showed the similar effect, in which MAN-triggered LC3 upregulation was blocked by NAC (**Figure 5D**). Finally, we turned to explore the mechanism behind this. As shown in **Figure 5E**, MAN decreased the phosphorylated mTOR and S6 expression levels but it was reversed in NAC plus MAN treated cells, suggesting that ROS generation activates autophagy through suppressing the AKT/mTOR pathway. In addition, cell morphology showed that NAC significantly reduced the cell death triggered by MAN (**Figures 5F, G**), suggesting that ROS is the cause of apoptosis. All these results revealed that ROS generation by MAN is the initiator of autophagy and apoptosis in lung cancer cells.

### Impairment of Autophagy Reduces the Cytoxicity of MAN in NSCLC Cells

**Figure 6:**
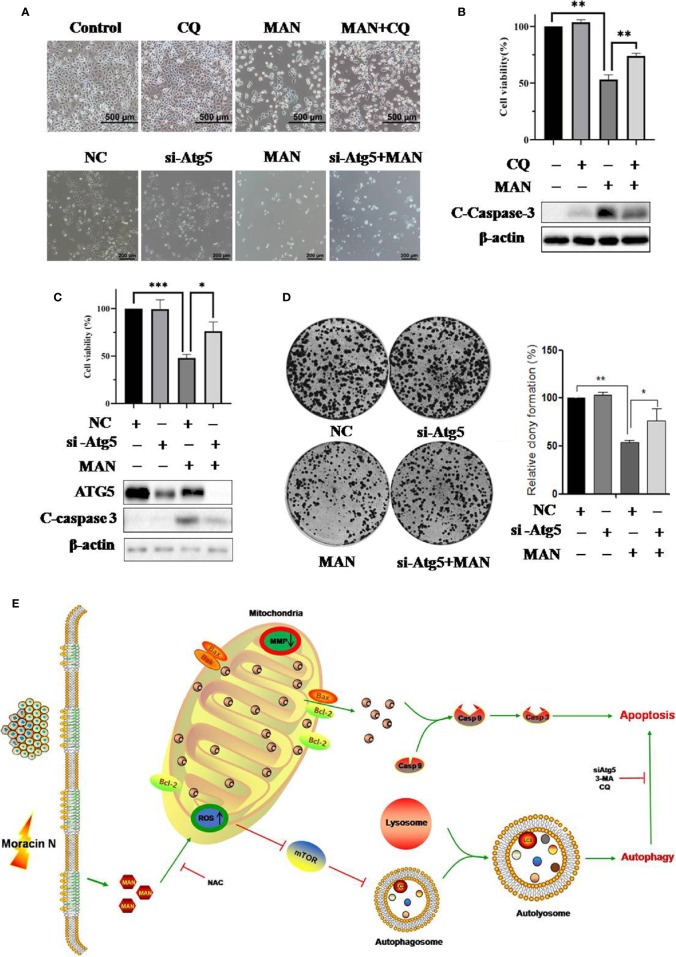
Impairment of autophagy reduces the cytotoxicity of Moracin N (MAN) in non-small-cell lung carcinoma (NSCLC) cells. **(A)** CQ or si-Atg5 was used to block autophagy in A549 cells. The cell morphology was observed under optical microscope (scale bar 100 μm) under MAN treatment (30 μM, 48 h). **(B, C)** as in(A), A549 cells were treated with MAN (30 μM, 48 h) under CQ treatment or Atg5 knockdown. Then cell viability was measured by MTT assay. * *p* < 0.05 ** *p* < 0.01 *** *p* < 0.01. Meanwhile, A549 cells were harvested for western blotting to detect ATG5 and caspase 3 protein levels. β-actin was used as a loading control. **(D)** A549 cells were first transfect with non-specific siRNA or siRNA specific for Atg5. After 72 h, cells were treated with MAN (30 μM) for 48 h and then reseeded into 6-well plates with a density of 500 cells per well for another 14 days to form clonies. The number of clonies were counted by Image J and statistically analyzed. * *p <*0.05 ** *p* < 0.01. **(E)** An illustrative model of MAN-induced autophagy and apoptosis in lung cancer.

To elucidate the role of autophagy in MAN-induced cell death, chloroquine (CQ) or siRNA for Atg5 was used to inhibit autophagy. Surprisingly, cell morphology results showed there was less cell death in MAN-treated cells under CQ treatment or Atg5 knockdown (**Figure 6A**). MTT assay demonstrated the similar results, in which the decrease of cell viability by MAN was attenuated (**Figures 6B, C**), suggesting that autophagy serves as cell death. Consistently, lower levels of cleaved caspase 3 by MAN was detected in Atg5 knockdown or CQ treated cells (**Figures 6B, C**). Moreover, clony formation assay showed that Atg5 knockdown attenuated the inhibitory effect of MAN on clony formation (**Figure 6D**), confirming that autophagy contributed to cell death.

## Discussion

MAN is a novel benzofuran derivative extracted form the leaves of *Morus alba* L. Previous study showed that MAN inhibits cancer cell proliferation (Tu et al., 2019). However, little study reveals the molecular mechanism. In this study, we put forward for the first time that MAN triggeres apoptosis amd autophagy through ROS accumulation. On the one hand, ROS generation induces mitochondria dysregulation to activate cell apoptosis. On the other hand, ROS production leads to the inhition of AKT/mTOR pathway and autophagy induction. One interesting finding is that blocking autophagy significantly reduces cell death, implying that autophagy play a critical role as cell death mechanism in MAN treated-lung cancer cells.

Targeting mitochondria is thought to be promising therapeutic approaches for cancer (Azad et al., 2009). Mitochondria is the potential target organelle of polyphenols (Hockenbery and Mutagenesis, 2010; Pan et al., 2016; James et al., 2018). Several reports have shown that polyphenols can stably accumulate in mitochondria, regulating the function of mitochondria-related proteins (Fiorani et al., 2010; Martínez-Pérez et al., 2016; Pan et al., 2016). Polyphenols, including genistein, biochanin A and xanthohumol, have been confirmed to have effect on mitochondrial electron transport chain (METC) and Bcl-2 protein family, leading to mitochondrial apoptosis (Carrasco-Pozo et al., 2012; Bi et al., 2018; Hsu et al., 2018). Hence, we decide to investigate the mitochondria changes after MAN treatment. We found that MAN significantly enhanced the Bax/Bcl-2 ratio, mitochondria matrix fragmentation and the decrease of MMP, indicating the dysregulation of mitochondria (**Figure 2**). Subsequently, cytochrome c was released into cytoplasm, followed by activation of caspase cascade (**Figure 2E**), suggesting that MAN induced mitochondrial-dependent apoptosis. As is known, mitochondria is an important source of ROS production as well as the main target of ROS function (Zhang et al., 2015). In our study, a dose-dependent ROS generation was observed in MAN-treated cells (**Figure 5A**). NAC treatment obviously decreased ROS levels and caspase 3 cleavage by MAN (**Figure 5**).

Autophagy is a conserved process involving in proteins, organelles degradation and recycling (Wang et al., 2017). Macroautophagy is characterized with conversion of light chain 3-I (LC3-I) to LC3-II , autophagosome formation (Mizushima et al., 2008; Mizushima and Levine, 2010; Zhang et al., 2012). Previous studies have noted that polyphenols can regulate autophagy (Nabavi et al., 2018). Thus, we aimed to examine the autophagy level changes by MAN. We found that MAN increased the levels of LC3-II and the formation of GFP-LC3 puncta (**Figure 3**). Meanwhile, lysosomal inhibitor CQ was applied to assess autophagic flux. We observed a further increase in LC3-II (**Figure 3**), suggesting the induction of autophagy flux by MAN. Lysosomal activation is proved to be important for autophagosomes fusion with lysosomes (Chang C. T. et al., 2017). Our results showed an enhanced lysosomal degradation as well as the promotion of autolysosome formation (**Figure 4**). These data indicated that MAN activates autophagy in A549 and PC9 cells.

There are various cellular signalling pathways regulating autophagy, including mTOR, PTEN and AMPK (Zhou et al., 2013; Liu et al., 2018a; Tomas-Hernández et al., 2018). Among them, mTOR signaling pathway is the most important mechanism (Zhou et al., 2013; Gao et al., 2018; Liu et al., 2018b). Our results showed that MAN treatment decreased the expression levels of phosphorylated-AKT, -mTOR, and -S6 (**Figure 3G**), demonstrating that AKT/mTOR pathway is responsible for MAN-triggered autophagy. ROS has also been reported to activate autophagy (Zhao et al., 2018). In our study, ROS inhibitor NAC reversed the induction of LC3-II and the formaiton of GFP-LC3 puncta by MAN (**Figure 5**). It could be attributed to the reactivation of AKT/mTOR pathway in the preasence of NAC, indicating that ROS generation activates autophagy.

The role of autophagy in cancer therapy is controversial. Some reports note that autophagy contributes to drug resistance and autophagy inhibition can enhance the cytotoxicity of paclitaxel, cisplatin and docetaxel (Kubisch et al., 2013; Mi et al., 2016; Datta et al., 2019). On the other hand, there are compounds reported to induce autophagy-mediated cell death in several types of cancer cells, such as naphthazarin (Pan et al., 2014), Flavokawain B (Luo et al., 2019), vitamin D and its analog EB 1089 (Acharya et al., 2011). In our study, autophagy inhibitors CQ or small interfering RNA for Atg5 were used to impair autophagy at different stage to investigate the functional role of autophagy. Interestingly, autophagy inhibition revesered the decrease of cell viability and caspase activation (**Figure 6**). Our work support that autophagy serves as cell death.

MAN is a natural polyphenols extracted from the leaves of *Morus alba* L. Extensive investigations have shown the anti-oxidantactivity of polyphenols and their derivative (Matsunaga et al., 2009; Filippopoulou et al., 2017). One recent research demonstrated that MAN has better anti-oxidant activity than resveratrol (Tu et al., 2019). However, in cancer cells, polyphenols were revealed to induce ROS generation (Das et al., 2010; Gibellini et al., 2010; Hu et al., 2019). In our study, MAN treatment significantly increased ROS accumulation in a dose-dependent manner either in A549 or PC9 cells (**Figure 5A**), which is in contradiction with its anti-oxidant activity. This could be related with the used dosage of polyphenols which hcan be pro-oxidant at a low concentration while it shows anti-oxidant effect at a high concentration (Shin et al., 2007). Another possible explanation may be due to the activity of peroxidase, one of the anti-oxidant enzymes which can eliminate free radicals. As is known, ROS is mainly produced by mitochondrial electron transport chain and peroxidase in cells. In some tumors, peroxidase is highly expressed compared with normal tissue (Gilabert et al., 1986; Chang et al., 2007; Park et al., 2013; Kwon et al., 2015). Peroxidase can catalyze polyphenols with phenol ring and some dietary phenolics oxidation to phenoxyl radicals, which eventually co-oxidizes with GSH forming ROS (Galati and O'Brien, 2004). In this process, ROS production is increased while GSH, the major intracellular anti-oxidant, is also consumed. These could be the reasons for the opposite effect of MAN on ROS in different cells. In human body, MAN has a good anti-oxidant effect (Tu et al., 2019); while it triggeres ROS generation in lung caner cells.

In summary, our research presents for the first time the effect of MAN on autophagy induction and mitochondrial apoptosis in human NSCLC (**Figure 6E**). ROS is revealed to be responsible for caspase activation and the AKT/mTOR pathway inhibition. In addition, MAN induced-autophagy serves as cell death. Thus, our findings demonstrate the great potential of MAN in anti-lung cancer and MAN might be developed to a novel therapy agent for NSCLC treatment in the future.

## Data Availability Statement

The raw data supporting the conclusions of this article will be made available by the authors, without undue reservation, to any qualified researcher.

## Author Contributions

CG, HY, and ZW performed the experiments of autophagosome formation and AKT-mTOR signaling pathway by MAN. XS, HTH, YS, MX, HLH and RG conducted the experiments of cell death and clony formation assay by MAN. JZ revised the manuscript and did the submission. SL did statistical analysis of most experiments. JT isolated and identified the novel secondary metabolite MAN from the root bark of *Morus alba* L.

## Conflict of Interest

The authors declare that the research was conducted in the absence of any commercial or financial relationships that could be construed as a potential conflict of interest.
